# Hypereosinophilic Syndrome, Multiorgan Involvement and Response to Imatinib

**DOI:** 10.7759/cureus.8493

**Published:** 2020-06-07

**Authors:** Caroline K Hana, Humberto Caldera

**Affiliations:** 1 Internal Medicine, University of Miami, Miami, USA; 2 Hematology/Oncology, Hematology/Oncology Associates, Palm Beach, USA

**Keywords:** imatinib, myeloproliferative neoplasm, hypereosinophilic syndrome, eosinophilic cystitis, eosinophilic cholecystitis, eosinophilic pancreatitis

## Abstract

Hypereosinophilic syndrome (HES) is an uncommon syndrome characterized by peripheral blood eosinophils count of more than 1,500/mm^3^ with associated tissue damage. It can be either primary or secondary, for example, due to parasitic infections or inflammation.

We present a case of a 49-year-old Asian female with recurrent hospital admissions for cholecystitis, gastritis, urinary cystitis, and pancreatitis. Her peripheral blood count showed excessive eosinophils 15,600-19,000/mm^3^ on different occasions. Pathology of her gallbladder and her gastric biopsies showed eosinophilic infiltration. Her bone marrow biopsy showed a normocellular marrow with active trilineage hematopoiesis, eosinophilia, mild megakaryocytic hyperplasia with a few atypical forms, and mild T-cell lymphocytosis. Flow cytometry showed no evidence of acute leukemia, or T-cell or B-cell lymphoproliferative disorder. On fluorescent in-situ hybridization (FISH), myeloproliferative neoplasms (MPN) testing was negative for platelet-derived growth factor receptor-alpha (PDGFRA), platelet-derived growth factor receptor-beta (PDGFRB), and fibroblast growth factor receptor-1 (FGFR1) rearrangement. Despite not having the FIP1L1‐PDGFRA (factor interacting with PAPOLA and CPSF1-platelet-derived growth factor receptor, alpha polypeptide) gene fusion, our patient responded to the treatment with a significant decrease in her absolute eosinophils count and resolution of her symptoms.

## Introduction

Hypereosinophilia is defined by an absolute eosinophil count (AEC) greater than 1,500/mm^3^ on two consecutive occasions, being persistent for a minimum of one month. When hypereosinophilia is associated with eosinophils induced end-organ damage, it constitutes the hypereosinophilic syndrome (HES) [1-3]. In light of some limitations of this definition, in 2010, revision of HES criteria for diagnosis recommended that the AEC of >1,500/mm^3^ not be a requirement for HES diagnosis [4].

From an etiologic point of view, hypereosinophilia disorders can be either primary, secondary, or idiopathic. The World Health Organization (WHO) classification of primary hypereosinophilia, also known as clonal or neoplastic hypereosinophilia, categorizes it to either chronic eosinophilic leukemia-not otherwise specified (CEL-NOS) or eosinophilia‐associated myeloid or lymphoid neoplasm. The latter is associated with rearrangement of platelet-derived growth factor receptor-alpha (PDGFRA), platelet-derived growth factor receptor-beta (PDGFRB), fibroblast growth factor receptor 1 (FGFR1), or with Pericentriolar Material 1-Janus Kinase 2 (PCM1‐JAK) [1,5,6]. As such, clonal HES can accompany any one of the myeloid malignancies defined by the WHO, for example, myeloproliferative neoplasms (MPN) or myelodysplastic syndromes [6]. HES variants include myeloproliferative variants, T-lymphocytic variants, familial HES, associated HES, and organ restricted hypereosinophilic conditions [4,7].

The secondary or reactive hypereosinophilia can be due to parasitic infections or allergic reactions, for example [2].

On the other hand, idiopathic eosinophilia is a diagnosis of exclusion when secondary and clonal causes of eosinophilia are excluded [8]. When HES is not associated with end-organ damage, it is referred to as HES of undetermined significance (HES-US) [3,4].

HES is a rare condition with an age‐adjusted incidence rate of approximately 0.036 per 100,000 [8,9].

## Case presentation

We report a case of a 49-year-old female of Asian descent who presented to the emergency department with burning with urination, frequency, and sensation of incomplete bladder emptying. The patient has a history of acute cholecystitis diagnosed a few months earlier. At that time, she underwent cholecystectomy and pathology showed eosinophilic cholecystitis. After this, she had persistent abdominal pain; she underwent esophagogastroduodenoscopy with esophageal, gastric and duodenal sampling showing mild chronic gastritis. The patient’s peripheral blood count at that time was showing persistent eosinophilia. The total white blood cell (WBC) count was 21,900/mm^3^, while the AEC was 15,600/mm^3^. Work up for parasitic infections was all negative.

**Figure 1:**
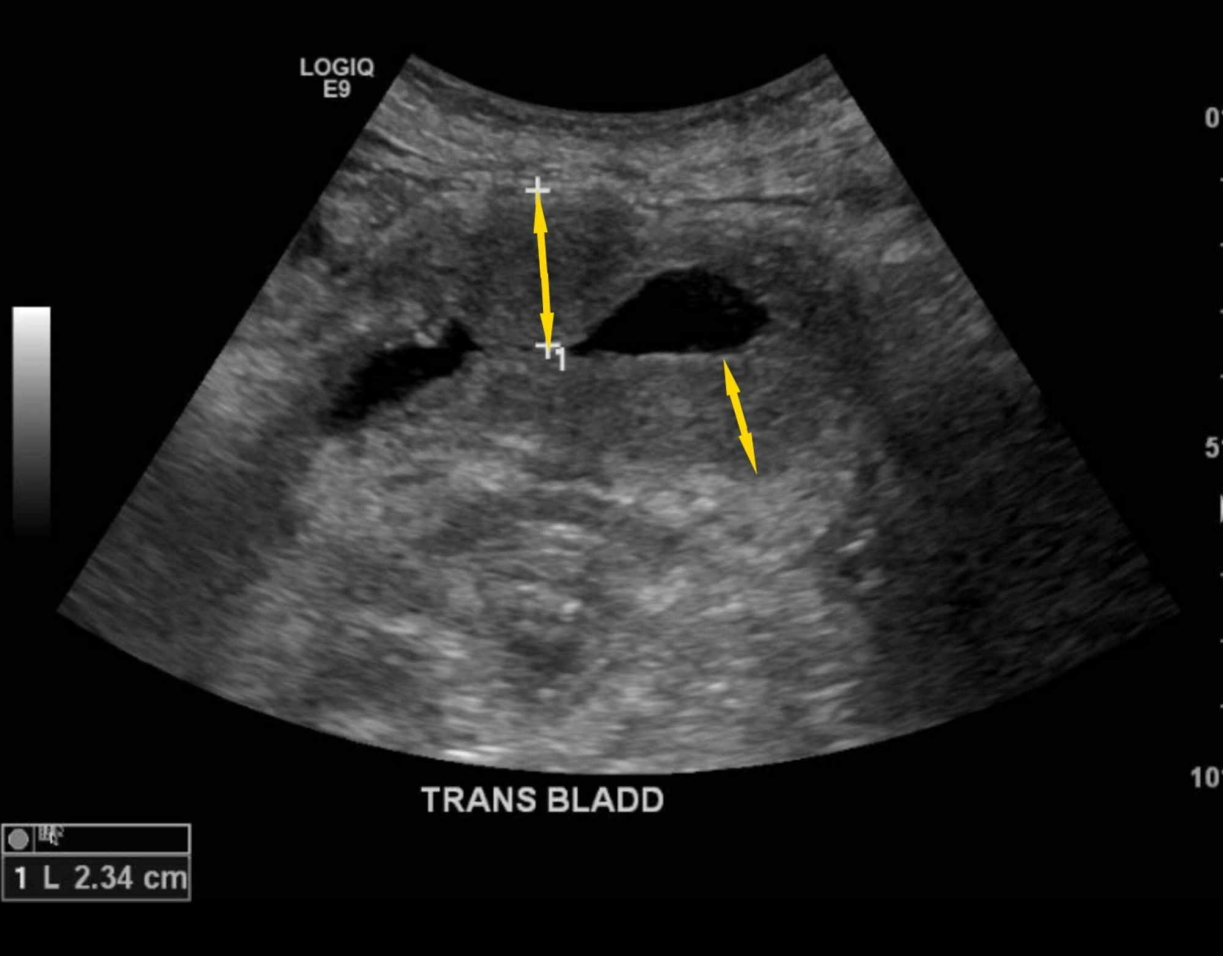
Ultrasound picture showing the bladder wall thickening

This presentation, the patient has been experiencing the burning and frequency symptoms for three weeks. Her symptoms were associated with lower abdominal pain. She denied any fever, bloody urine, nausea, or vomiting. She was diagnosed with urinary tract infection (UTI) as an outpatient and had received seven days of an unknown antibiotic, then was started on ciprofloxacin, which she administered for four days. However, her symptoms did not improve. On admission, the patient was afebrile and vitally stable. Her physical examination was noted for suprapubic tenderness. Her lab work showed elevated WBC of 25,000/mm^3^ with AEC of 19,000/mm^3^, hemoglobin of 14.4 mg/dL, and platelets of 301,000 cells/mm^3^. Urinalysis was positive for nitrites, protein > 500 mg/dL and RBCs > 100/HPF. The patient was admitted for complicated cystitis with sepsis. Ultrasound of the pelvis showed a trace amount of free fluid in the pelvis with diffuse wall thickening of the bladder (Figure 1).

**Figure 2:**
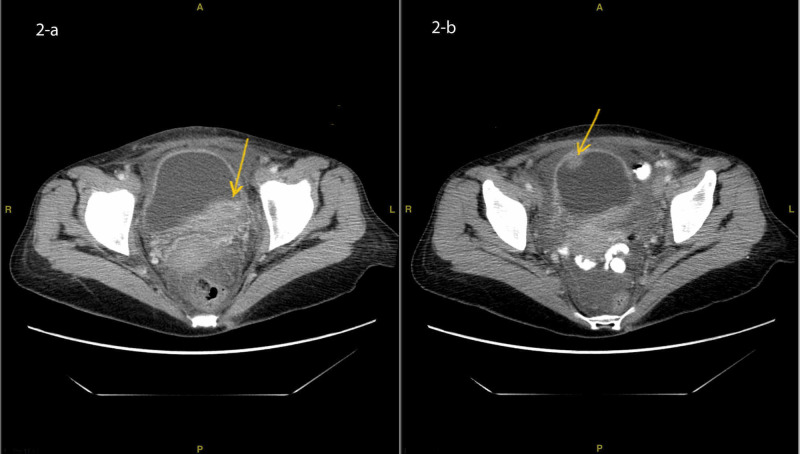
CT scan of the abdomen and pelvis showing the posterior 2.5 x 6.6 cm bladder mass (a) and anterior 1.3 x 2.1 cm bladder mass (b)

CT scan of the pelvis with oral and IV contrast showed multiple urinary bladder masses, the largest of which measured 2.5 x 6.6 cm with an anterior wall mass 1.7 x 2.2 cm (Figure 2 (a, b)).

The patient was started on ceftriaxone for complicated UTI. Cystoscopy showed a bladder mass, which was resected. Pathology showed benign polyploid urothelium with reactive changes and extensive eosinophilic infiltrate, numerous histiocytes with eosinophilic crystal-like structures; there was no evidence of malignancy. The patient was then treated with prednisone 20 mg, which she continued for two months. The patient’s peripheral blood count showed a response to prednisone with a WBC count of 6,000 cells/mm^3^ and AEC of 300 cells/mm^3^. However, she continued to have urinary symptoms; hence, she was started on mirabegron for overactive bladder. 

The patient returned for right-sided abdominal pain with tenderness on palpation. Lab work showed a WBC count of 7,800 cells/mm^3^, AEC 1903 cells/mm^3^, lipase 282 U/L, and amylase 144 U/L. CT scan of the abdomen and pelvis showed ill-defined fat stranding of the peripancreatic fat with no pancreatic masses or peripancreatic fluid. Moderate right and mild left hydronephrosis were also noted. No hepatomegaly or splenomegaly was detected.

Given her eosinophilia, the patient underwent bone marrow biopsy, which showed normocellular marrow with active trilineage hematopoiesis, eosinophilia, mild megakaryocytic hyperplasia with a few atypical forms and mild T-cell lymphocytosis. Flow cytometry showed no evidence of acute leukemia, or T-cell or B-cell lymphoproliferative disorder. FISH myeloproliferative neoplasm testing was negative for PDGFRA, PDGFRB, and FGFR1 rearrangement. Polymerase chain reaction (PCR)-based advanced sequencing assay of her deoxyribonucleic acid (DNA) did not detect any of the following mutations: Janus kinase-2 (JAK2)-V617F, calreticulin (CALR) exon 9, JAK2 exon 12, myeloproliferative leukemia (MPL) exon 10, or the receptor for colony-stimulating factor 3 (CSF3R) 14/17.

Tryptase level was within normal limits. However, her B12 level was elevated at 1,453 ng/ml.

She was started on a second course of prednisone; however, a month later, she developed abdominal pain again, and her CT scan showed ileo-ileal intussusception within the right lower quadrant with no evidence of bowel obstruction. Repeat peripheral blood counts showed WBC 11,300 cells/mm^3^ and AEC 4,700 cells/mm^3^. A higher dose of prednisone resulted in the patient having significant gastrointestinal upset and anxiety.

Since she was not responding to prednisone, and given the significant toxicity of interferon, she was started on imatinib, a tyrosine kinase inhibitor, at 400 mg daily. She received the treatment for two weeks and then stopped it due to developing side effects, which included periorbital edema, joint pain, rash, and fluid retention. Despite not having the FIP1L1‐PDGFRA gene fusion, our patient responded to the treatment with a decrease in her absolute eosinophils count from 4,700/mm^3^ to 700/mm^3^. On her six-month follow-up, the patient had no fever or other signs of inflammation. 

## Discussion

HES is a rare syndrome characterized by AEC of >1,500 cells/mm^3^ and with evidence of end-organ damage. According to the WHO classification of eosinophilic disorders, the idiopathic type is diagnosed after the exclusion of primary and reactive eosinophilias [8].

The hematologic profile of patients with HES shows a mean peak AEC of 6,600/mm^3^ with a range of 1,500-400,000/mm^3^ [10]. Traditionally, peripheral blood eosinophilia has been divided into mild (500-1,500 cells/mm^3^), marked (>1,500 cells/mm^3^), and massive (>5,000 cells/mm^3^) eosinophilia [3].

In our case, the patient had persistent eosinophilia for more than three years. She had evidence of end-organ damage with biopsy-proven eosinophilic cholecystitis, urinary bladder cystitis/masses, gastritis, pancreatitis, and perihepatic abscess. In one case series that investigated the incidence of different organs involvement, anemia was present in 53% of patients, thrombocytopenia was more common than thrombocytosis (31% vs. 16%), and bone marrow eosinophilia ranged from 7% to 57% (mean 33%) [11]. Only one case reported eosinophilic cystitis [12].

Organ damage induced by HES is due to the eosinophilic infiltration of the tissues accompanied by the mediator release from the eosinophil granules. Hence, the level of eosinophilia is not a true reflection of organ damage [8]. In general, eosinophils movement to the tissues is mediated by various eotaxins, for example, interleukin-5 (IL-5), or other chemo-attractants, for example, complement anaphylatoxins C3a and C5a. After recruitment, eosinophils cause tissue damage by generating oxidative stress through eosinophil peroxidase (EPO) [13] and through their major basic protein (MBP), which exerts cytotoxic effects by interfering with the electrical homeostasis of the cell surface. MBP is also a trigger for mast cell degranulation [14]. Eosinophils may secrete their products selectively and differentially, in response to specific ligands, but the exuberant release of granule-derived proteins, by degranulation or cytolysis, may contribute to the tissue damage associated with eosinophil-associated diseases [15].

Despite having elevated B12 level, our patient had no other clinical features of the myeloproliferative variant of HES with no evidence of anemia or thrombocytopenia, no hepatomegaly or splenomegaly, and no chromosomal abnormalities.

Regarding the genetic profile of our patient, she did not have JAK, CALR, MPL or CSF3R making a clonal primary eosinophilia unlikely. She also had no BCR-ABL mutation. Having a normal tryptase level excluded the possibility of mastocytosis, her FISH MPN was negative for PDGFRA, PDGFRB, and FGFR1 rearrangement. These tests would support the diagnosis of idiopathic HES [16].

The mainstay for the treatment of idiopathic HES is corticosteroids. However, other treatment options include hydroxyurea and interferon-alpha [1]. Some cases have reported a good response to etoposide [17]. Imatinib has been a well-established treatment for patients with FIP1L1-PDGFR alpha rearrangement. Few studies showed that patients without this rearrangement should also be given a trial of imatinib [18,19].

This is an interesting case as it shows HES causing multiorgan involvement at different anatomic levels, including gastrointestinal and genitourinary systems. This case also highlights the limited treatment options for patients with idiopathic hypereosinophilia syndrome and an excellent response to imatinib. Our patient was not a candidate for steroids given the partial response at lower doses and the severe side effects at higher doses. Hence, she was deemed a candidate for imatinib given the recurrent and multiorgan involvement. It is worth noting that the patient was followed for six months, which may represent a temporary response; however, this may also represent diagnostically occult PDGFRA or PDGFRB mutations, or other unknown pathogenic targets [18,19].

## Conclusions

HES is an uncommon syndrome characterized by AEC > 1,500/mm^3^ with tissue damage. Workup should include tests to rule out primary and secondary causes, to establish the diagnosis of idiopathic HES and explore the cytogenetic background. Treatment of this group of diseases maybe sometimes challenging. Imatinib has been a well-established treatment for HES with FIP1L1-PDGFR alpha rearrangement. The response to imatinib in patients who lack this mutation have been documented in few studies; however, further studies with longer follow-up are required to elaborate whether this is a temporary response, a diagnostically occult PDGFRA or PDGFRB mutations, or other unknown pathogenic targets. Besides, whether the prior use of steroids in our case has contributed to her response to imatinib is also unclear.

## References

[1] Shomali W, Gotlib J (2019). World Health Organization-defined eosinophilic disorders: 2019 update on diagnosis, risk stratification, and management. Am J Hematol.

[2] Leru PM (2019). Eosinophilic disorders: evaluation of current classification and diagnostic criteria, proposal of a practical diagnostic algorithm. Clin Transl Allergy.

[3] Valent P, Klion AD, Horny HP (2012). Contemporary consensus proposal on criteria and classification of eosinophilic disorders and related syndromes. J Allergy Clin Immunol.

[4] Simon HU, Rothenberg ME, Bochner BS (2010). Refining the definition of hypereosinophilic syndrome. J Allergy Clin Immunol.

[5] Bain BJ (2010). Myeloid and lymphoid neoplasms with eosinophilia and abnormalities of PDGFRA, PDGFRB or FGFR1. Haematologica.

[6] Tefferi A, Gotlib J, Pardanani A (2010). Hypereosinophilic syndrome and clonal eosinophilia: point-of-care diagnostic algorithm and treatment update. Mayo Clin Proc.

[7] Gleich GJ, Leiferman KM (2009). The hypereosinophilic syndromes: current concepts and treatments. Br J Haematol.

[8] Gotlib J (2017). World Health Organization-defined eosinophilic disorders: 2017 update on diagnosis, risk stratification, and management. Am J Hematol.

[9] Crane MM, Chang CM, Kobayashi MG, Weller PF (2010). Incidence of myeloproliferative hypereosinophilic syndrome in the United States and an estimate of all hypereosinophilic syndrome incidence. J Allergy Clin Immunol.

[10] Ogbogu PU, Bochner BS, Butterfield JH (2009). Hypereosinophilic syndrome: a multicenter, retrospective analysis of clinical characteristics and response to therapy. J Allergy Clin Immunol.

[11] Flaum MA, Schooley RT, Fauci AS, Gralnick HR (1981). A clinicopathologic correlation of the idiopathic hypereosinophilic syndrome. I. Hematologic manifestations. Blood.

[12] Jiang P, Wang C, Jin B, Lin Y, Chen S (2014). Eosinophilic cystitis in a patient with hypereosinophila syndrome: a case report. Exp Ther Med.

[13] Ramirez GA, Yacoub MR, Ripa M (2018). Eosinophils from physiology to disease: a comprehensive review. Biomed Res Int.

[14] Diny NL, Rose NR, Čiháková D (2017). Eosinophils in autoimmune diseases. Front Immunol.

[15] Roufosse F, Weller PF (2010). Practical approach to the patient with hypereosinophilia. J Allergy Clin Immunol.

[16] Hu Z, Boddu PC, Loghavi S (2018). A multimodality work-up of patients with hypereosinophilia. Am J Hematol.

[17] Razaq W, Beautyman E (2009). Successful treatment of refractory idiopathic hypereosinophilic syndrome with etoposide. Am J Ther.

[18] Helbig G, Hus M, Halasz M (2012). Imatinib mesylate may induce long-term clinical response in FIP1L1-PDGFRalpha-negative hypereosinophilic syndrome. Med Oncol.

[19] Butterfield JH (2009). Success of short-term, higher-dose imatinib mesylate to induce clinical response in FIP1L1-PDGFRalpha-negative hypereosinophilic syndrome. Leuk Res.

